# Female gender as a risk factor for developing COPD

**DOI:** 10.17179/excli2021-4118

**Published:** 2021-08-06

**Authors:** Shakti D. Shukla, Madhur D. Shastri, Niraj Kumar Jha, Gaurav Gupta, Dinesh Kumar Chellappan, Tanmay Bagade, Kamal Dua

**Affiliations:** 1 Discipline of Pharmacy, Graduate School of Health, University of Technology Sydney, Ultimo, NSW 2007, Australia; 2 Faculty of Health, Australian Research Centre in Complementary and Integrative Medicine, University of Technology Sydney, Ultimo, NSW 2007, Australia; 3 School of Pharmacy and Pharmacology, University of Tasmania, Hobart, Australia; 4 Department of Biotechnology, School of Engineering & Technology (SET), Sharda University, Greater Noida, 201310, UP, India; 5 School of Pharmaceutical Sciences, Jaipur National University, Jagatpura, 302017, Jaipur, India; 6 Department of Life Sciences, School of Pharmacy, International Medical University, Bukit Jalil, Kuala Lumpur 57000, Malaysia; 7 School of Medicine and Public Health, Faculty of Health and Medicine, University of Newcastle, Callaghan, NSW, 2308, Australia

## ⁯⁯⁯

***Dear Editor,***

Chronic obstructive pulmonary disease (COPD) is a progressive lung disease with an increasing burden and associated mortality globally (Soriano et al., 2020[16]). The primary risk factors include cigarette smoking, although any long-term exposure to hazardous chemicals, dust and fumes could result in the initiation, development, and/or severity of COPD (Soriano et al., 2020[16]). The Global Burden of Disease report estimated the number of daily smokers was as high as 1.14 billion across 195 countries and territories in 2019, who consumed more than 7.41 trillion cigarette-equivalents of tobacco (Reitsma et al., 2021[14]). Indeed, smoking cessation is presently the most effective intervention that significantly slows the progression of COPD and reduces the disease severity (Tashkin, 2015[17]). In addition to cigarette smoking, both indoor air pollution (household air pollution from burning solid fuels for cooking or heating) and outdoor air pollution (particulate matter) represented the predominant risk factor for developing COPD among women, especially in low- and low-middle income countries (Soriano et al., 2020[16]). Out of 4.2 million deaths globally due to indoor air pollution, 22 % die due to COPD, with a significantly higher prevalence among women compared to men (Austin and Mejia, 2017[2]; Apte and Salvi, 2016[1]). 

COPD has been considered a disease primarily associated with male gender, albeit this notion mainly was because more men smoked regularly than women, although the smoking prevalence in women was 28 % (Jafari et al., 2021[11]). Also, empirical data points to the fact that men usually commenced smoking earlier than women (by several decades) (Petty, 2006[12]). However, mounting epidemiologic data highlights that females may be significantly more susceptible than males to the harmful effects of cigarette smoke and potentially more prone to COPD development. In fact, women who smoke are 50 % more likely to develop COPD than men (Barnes, 2016[3]). In terms of the role of gender and exposure to cigarette smoke on the development of COPD, the results from two large populations, i.e., the Copenhagen City Heart Study (CCHS) and the Glostrup Population Studies (GPS) (n=13,897), with a relatively prolonged follow-up (7-16 years), reported that smoking indeed exerted a greater reduction in lung function of females than males. Notably, the risk of hospitalization for COPD was also found to be higher in females than in males (Prescott et al., 1997[13]). 

The data to date does point to differential susceptibility to developing COPD in males and females who smoke regularly. A systematic review and a meta-analysis of 11 studies (n=55,709) demonstrated that women smokers have a more considerable decline in lung function. This was particularly significant in women who were in the age range of 45-50 years (Gan et al., 2006[9]); the rate of reduction being 0.98 % per pack-year in men versus 1.21 % per pack-year for women (Dransfield et al., 2006[8]). The age of 45 years also seems to be critical in most individuals in determining whether smoking would result in the development of COPD or not. Thus, it seems likely that women do possess a higher risk of developing COPD than men. Similarly, Sørheim et al. also assessed both low and high smoke exposure subgroups and reported that women demonstrated a relatively lower forced expired volume in one second (FEV_1_) than men (48.7 % predicted for women vs 55.8 % predicted for men). Moreover, females also had a higher prevalence of more severe COPD as assessed by GOLD COPD stage (III and IV (Sørheim et al., 2010[15]). The effects of currently smoking status in females also spanned to the overall lung pathology, as assessed by CT lung density, which more rapidly declined in females than males in ECLIPSE study (n=1928) (Coxson et al., 2013[6]). 

The clinical presentation of COPD also varies significantly among men and women. For instance, females report greater symptoms, including shortness of breath, depression, and lower health-related quality of life. Notably, females demonstrated more airway predominant disease phenotype (DeMeo et al., 2018[7]) compared to men.

The possible explanation for the disparity between smoking and gender regarding their susceptibility to developing COPD could be due to several factors. Firstly, the hormones could play a key role; however, the effects of hormones on the development of smoking-related COPD are not understood completely and debatable. For instance, one study found that hormone replacement therapy does not affect the overall incidence of COPD in women (Barr and Camargo, 2004[4]), whilst another study showed that women on hormone replacement therapy demonstrated marked improvements in the lung function (Carlson et al., 2001[5]). Secondly, and perhaps more likely reason relates to the difference in the size of the lungs between men and women. Seemingly, the lungs of women are smaller in size, thus, the oxidative stress and damage caused by smoking could be pronounced in women than men even despite comparable smoking histories (Prescott et al., 1997[13]); however, the mechanisms are yet to be fully elucidated. 

Gender bias in medicine and medical research is another area that needs urgent global attention. The assumption that 'what works for men also works for women' is often misleading and some authors consider it as a fundamental flaw of the evidence basis of medicine (Holdcroft, 2007[10]). Although women are 50 % of the world population, they are under-diagnosed and under-researched in studies related to COPD. This might be partly due to the strict ethical requirements of including women in clinical trials, making it a time-consuming recruitment process. The wide variation of hormonal changes due to menstruation, age, use of hormonal contraception, or reproductive state of women further complicate the task of interpreting results of studies that include female participants in medical research related to COPD. 

It is imperative that these gender differences are accounted for when assessing the progression or severity of COPD for personalized management plan of the disease. Furthermore, focused studies are warranted to tease out the potential diagnostic, therapeutic and prognostic implications of gender on COPD initiation/progression, which could certainly be crucial for the effective management of female patients with airway diseases.

## Notes

Shakti D. Shukla and Kamal Dua (Discipline of Pharmacy, Graduate School of Health, University of Technology Sydney, Ultimo, NSW 2007, Australia; E-mail: Kamal.Dua@uts.edu.au) contributed equally as corresponding author.

## Conflict of interest

The authors have no conflict of interests to declare.

## Funding

None.
